# Assessing policy-makers’, academics’ and experts’ satisfaction with the performance of the Palestinian health research system: a qualitative study

**DOI:** 10.1186/s12961-018-0341-x

**Published:** 2018-07-25

**Authors:** Mohammed AlKhaldi, Yehia Abed, Constanze Pfeiffer, Saleem Haj-Yahia, Abdulsalam Alkaiyat, Marcel Tanner

**Affiliations:** 1 Swiss Tropical and Public Health Institute, Socinstr, 57, 4002 Basel, Switzerland; 2 University of Basel, Petersplatz 1, 4003 Basel, Switzerland; 3 Faculty of Public Health, Al-Quds University, Jerusalem, Palestine; 4 University Teaching Hospital, An-Najah National University, Nablus, Palestine; 5 Faculty of Medicine and Health Sciences, Najah National University, Nablus, Palestine; 6 School of Clinical Sciences, University of Bristol, Bristol, United Kingdom

**Keywords:** Health experts, Satisfaction, Health research system performance, Palestine

## Abstract

**Background:**

There is a growing demand within international health agencies to ensure health research systems (HRSs) are strengthened and well-functioning to support healthcare systems (HCSs). Understanding HRS performance through system actors is an indispensable move in analysing this system. This study aims to examine policy-makers’, academics’ and experts’ satisfaction with overall HRS performance, while also investigating their perceptions about political will and attention towards health research. Ultimately, we want to identify gaps related to performance and generate insights on how to move forward for HRS performance strengthening.

**Methods:**

This study was carried out in Palestine, targeting three sectors, namely government institutions, public health universities, and major local and international health non-governmental organisations (NGOs). Semi-structured, in-depth interviews (IDIs) and focus group discussions (FGDs) were conducted with participants. The institutions from the three sectors were selected based on stated criteria and peer reviews. Data were translated from Arabic into English, transcribed, content checked by the principal investigator, imported to a software programme (MAXQDA 12), and then coded. Thematic content analysis was used.

**Results:**

A total of 104 experts participated in 52 IDIs and 52 experts participated in 6 FGDs. Findings revealed three principal domains. First, the HRS in Palestine is remarkably underperforming, and the majority of experts were unsatisfied. Participants perceived the system as ineffective and inefficient, poorly managed and lacking systematic assessment. Second, the factors behind system underperformance were (1) an unstructured system and the lack of a research culture as well as of a governing body or policies; (2) health research was seen as individualistic, non-development driven and unutilised in policy decisions; and (3) considerably deficient coordination and essential resources. The third finding showed inadequate political support and engagement, which then also related to system underperformance.

**Conclusions:**

The Palestinian HRS is perceived as underperforming by health experts at different levels, where research is not on the leadership agendas. Potential actions should be taken to actively engage the state health decision-makers and inform them of the importance, uses and impacts of performance assessment. Findings urge policy-makers and legislators to build an inclusive and national body of governance with agreed strategies including fundamentally hybrid and aligned performance assessment mechanisms, such as a research observatory platform. In addition, it is recommended to establish a strategic plan to expand professionals’ research awareness and abilities, as well as empower the institution’s research monitoring and evaluation capacities.

**Electronic supplementary material:**

The online version of this article (10.1186/s12961-018-0341-x) contains supplementary material, which is available to authorized users.

## Background

Health research systems (HRS) form a key pillar of the structure of healthcare systems (HCSs), guiding them to deliver better health policies and services [1, 2]. Research performance in terms of functions and processes does not automatically contribute to better health action; the more useful issue is the process of knowledge generation in order to better understand health problems [3]. Therefore, developing effective and efficient HRS performance is an important step towards addressing society’s needs [4] and, consequently, understanding system performance is vital for strengthening it [5]. This is considered a priority in the context of international efforts to correct the 10/90 gap and to address various health research (HR) gaps [3, 6–8]. The starting point of HRS analysis is to have a clear picture of current HR, and the necessary development actions [9]. This first requires a deep understanding of the system actors’ perceptions, be they research funders, producers or users, to investigate HRS pillars, particularly assessing their performance and political commitment to HR. Palestine and the region have seen important improvements in research productivity while overall research performance is poor, with critical deficits in stewardship, capacity, translation and problems attributed primarily to both financial and political constraints [10–13].

It is difficult to assess the stewardship owing to the complexity of the HRS and the diversity of players and sectors [14], with multiple roles in managing and evaluating the system [15]. The journey from research production to evidence-based practice and health impacts is usually long, non-linear and multi-faceted [16]. These stages need to be thoroughly understood in order to identify what HRS does and how it performs and works [9, 17]. This study employs a system perspective as proposed by WHO and the Council on Health Research for Development (COHRED) [5, 9, 14, 18, 19] with its various conceptual analysis approaches. This study adopts those approaches that include key aspects needed to carry out such a performance assessment. This approach serves to both observe the system performance and its processes as well as to offer a platform from which actions for system improvements can be identified [9].

Based on perceptions analysis, this assessment comprises stakeholders’ satisfaction, a description of the actual status of HRS performance and political attention, and performance deficiencies and solutions identification during research financing, production or utilisation phases. Any system without systematic monitoring and evaluation is blind, and HRS performance is an essential element falling under the stewardship function [5]. Making this system performance effective means employing evidenced-based practices, while efficiency engages correct practices with valuable benefits at low costs [20]. In light of lacking standards or quantitative indicators to monitor and evaluate research and its societal benefits, this study fills an important knowledge gap because it focuses on performance and its deficiencies which are rarely addressed in Palestine and in the region because formal HRSs are lacking [21]. As used by other authors, this descriptive study employs a qualitative ‘snapshot’ assessment and a complementary approach of HRS performance analysis [5, 14, 18, 22, 23].

Any HRS has a wide range of stakeholders, who all have interests and influence on how research is defined, performed and used. Three relevant sectors have been purposively targeted in Palestine, namely government, academia, and local and international non-governmental organisations (NGOs). It is worth investigating the technical views of various actors in different sectors to understand the trends of their perceptions [15]. Moreover, varied perspectives on topics, such as satisfaction with system performance or political support to HR, allow the system to be understood from multiple angles, where actors suggest innovative ideas and strategies for application and strengthening interventions [24, 25].

This study is in line with the WHO strategic direction on research for health. It is necessary to fill the knowledge gap and demystify ambiguity on HRS performance and the political attention to HR in the face of literature scarcity and unrecognised status. This topic is not sufficiently addressed in the HRS analysis toolkit developed by WHO [18]. Locally, studies showed that the state of scientific research in Palestine is unclear, with a lack of orientation [26], and that HRSs in developing countries, including Palestine, are not systemically evaluated to high standards; hence, varying assessment methods to analyse HRS are important [14, 27]. Globally, evidence has emphasised that this topic is a challenge [5], and WHO also underlined inadequate system understanding and the fact that HR is not politically appreciated [3]. Another rationale for this study is that understanding the overall satisfaction with performance and the status of state attention to HR is the main entryway to a functioning system, conceptually and operationally [5], where awareness would be associated with practices. This understanding leads to a sustainable HRS by recognising trends in HRS and whether performance is improving or declining, and this may reveal whether the Palestinian political attention to a developmental vision of HRS is sufficient or negligent. A lack of understanding misleads the system, may create duplications and inefficiencies, and may also negatively affect the credibility of the produced research [24, 28]. The current study is part of a national research project aiming to generate an overview of the satisfaction level of the Palestinian health policy-makers, academics and experts on overall HRS performance and the political attention towards HR. Four objectives guide this study, namely (1) to understand stakeholders’ satisfaction with the overall performance pattern; (2) to examine the state of political government support and attention towards HR; (3) to identify the relevant performance gaps echoed by health policy-makers, academics and NGOs experts; and (4) to provide important implications and potential insights towards Palestinian HRS strengthening with regards to performance and political support.

## Methods

### Design

A qualitative cross-sectional descriptive situation analysis approach was used by conducting in-depth interviews (IDIs) and focus group discussions (FGDs) along with the application of an inductive approach. This study approach was adapted from international models developed by WHO and COHRED in investigating the perceptions of HRS stakeholders on performance and political attention, holistically from a systems perspective. Another reason for using this approach is that the system analysis relies on a systems thinking perspective and comprehensive understanding [18, 29]. In addition, using the national HRS assessment framework helps to provide principles for system analysis and ensures long-term sustainable development, firstly, because it is sensitive to limited resources and, secondly, because it integrates local experience and understanding into the national health research system improvement process [9, 18]. This design is appropriate in light of the complexity of HCS and the HR environment by helping to understand the research subject from numerous perspectives [30]. The study setting was Palestine, West Bank (WB) and Gaza Strip (GS), both areas being geographically separated (Additional file 1: Supplement 1). The study ran from January until July 2016. The targeted institutions in the three sectors (illustrated in Additional file 1: Supplement 2) were as follows:

1. Six bodies in the government sector: Ministries of Health (MOH), Higher Education, Finance and Planning, Palestinian Legislative Council, Palestinian Medical Council and Palestinian Central Bureau of Statistics.
2. The academic sector: health and medical faculties of 11 major universities and colleges in Palestine, and from Lebanon whose teams wrote intensively on the study subject. Selecting this expert is to grasp the subject from the local as well as the regional perspectives, and to get a complementary understanding from a relevant outsider perception.
3. Local and international NGOs: 10 international NGOs and 11 local Palestinian NGOs.

### Sampling and data collection

Purposive sampling was used. To reduce selection bias and to ensure knowledge saturation, active participation and adequate representation, mixed sampling was used through four strategies. First, is criterion sampling, to select participants who are able to provide particular information on certain topics under investigation. Secondly, critical case sampling was used to target experts who gave critical and factual information. Thirdly, snowball sampling determined other suitable participants that were not known to us at the onset of the study. The fourth sampling strategy was a homogenous group where participants from a similar background and with similar experience were brought together [31]. The initial list of potential participants across three sectors (government, academia and NGOs) was prepared based on the first author’s knowledge. He is a Palestinian with a background in public health, working for more than 9 years in the three sectors.

Participants were allocated to two groups, wherein 52 of the political key-informants were assigned to IDIs and 52 technical participants to FGDs, without double participation. Expert consultations and rigorous peer reviews were carried out to attain sample representation, and then participant’s lists were merged into one final list. Inclusion and exclusion criteria were established to clearly guide the selection process (Additional file 1: Supplement 3).

The study was designed with the diversity of participants’ levels of knowledge, experience and positions in mind. Potential participants were initially phoned and emailed by the principal investigator and provided a copy of the study information sheet. Participants who did not respond to the initial contact were called again and sent another email after a couple of weeks. Those who agreed to participate (104 experts) were assigned to participate in either IDIs or FGDs. Prospective participants received the full agenda and discussion outlines in advance via email, followed by invitation after a few days. A balanced selection of participants was achieved between WB & GS. Participants from executive political and front management levels of targeted HRS institutions were assigned to IDIs and participants from the middle technical and management level were assigned to FGDs.

For both IDIs and FGDs, semi-structured guides with open-ended questions were formulated according to the principles laid out in the relevant literature [1, 3, 5, 18, 19] (see Additional file 1: Supplement 4a for IDIs and 4b for FGDs). Both instruments focused on five themes, namely (1) HRS conceptualisation and its importance; (2) stakeholders satisfaction on HRS performance, which is the interest of this study; (3) governance, policy and financing; (4) stakeholders analysis, HRS capacities and research priorities in Palestine; and (5) HRS challenges and insights for strengthening.

To appraise trustworthiness and credibility, questions were discussed among the research team as well as with international scientists and local experts in Palestine. The questions were piloted in five IDIs and one FGD for clarity. Building on the pilot, both instruments were revised. The overall quality of this study is appropriate where a comprehensive model, internationally developed, was adopted along with a suitable design, a variety of methods and sampling, and a double check of the quality of data analysis and interpretation. These aspects were subjected to a rigorous and precise review by local and international experts. Moreover, for all relevant managerial levels and sectors, sample diversity and representation was achieved. However, it is noteworthy that a bias related to the political situation prevailing during the study period may have a relative effect on the outputs of the study.

Overall, 45 IDIs were performed face-to-face and 7 by Skype call due to movement restrictions in the field. IDIs ranged from 45 to 60 min; 18 academic interviewees were from different health schools, 20 interviewees were from government policy and decision-makers representing the 6 different bodies, and 19 experts were interviewed from 10 local agencies and 5 from the international agencies. Overall, 52 participated in 6 sectoral FGDs, 3 in WB and 3 in GS, only 1 FGD for each sector in both areas. Each FGD took approximately 90 minutes and included 6–10 persons. A trained research team coordinated and managed all data collection and the principal investigator guided all fieldwork.

### Data analysis

IDIs and FGDs were audio-recorded in Arabic and were translated and transcribed verbatim in English. Transcripts were revised manually by the principal investigator for precision, checked and cleaned for accuracy. The data was analysed using thematic content analysis [32]. Themes and codes were inductively established guided by the conceptual framework developed by the relevant HRS literature. Field notes were also used during data collection and analysis. MAXQDA 12 (VERBI GmbH, Berlin) software was used in the analysis. The first author analysed transcripts line by line and created codes based on emergent themes. Codes were reviewed and patterns of agreement and disagreement established.

## Results

### Sociodemographic characteristics of participants

From 38 institutions across the three sectors, 104 experts participated in both methods of inquiry, while 11 declined. The overall status of study participants is diverse and wide-ranging as HR is conceptually broad and interlinked [33]. The characteristics of IDI participants are illustrated in Additional file 1: Table S1, where the majority had a PhD with more than 20 years of experience, particularly NGOs. Participants and their institutions were distributed as follows: 18 experts from 8 academic institutions, 19 from 15 NGOs (10 local and 5 international), and 15 participants from 5 government institutions. The participants were from the first leadership levels. Additional file 1: Table S2 shows the 6 sectorial FGDs carried out (3 in the WB, 3 in the GS), with a total of 52 participants. About one-third of participants were female, and most participants were aged above 40 years old. The majority had postgraduate degrees with more than 10 years’ experience. Most FGD participants had more than 10 years’ experience.

### Concurrent experts’ overall satisfaction with HRS performance

Respondents’ overall satisfaction with HRS performance was remarkably inconsistent, falling into three categories, as dissatisfied, relatively satisfied and satisfied. While most participants were not satisfied with HRS, a few expressed their satisfaction. Government respondents were relatively satisfied. Most of them strongly indicated that HR performed seasonally, but not for developmental and institutional reasons. Moreover, other views from academia were not fully satisfied; there was an agreement that this system is neither well-performing nor effective and efficient. Two quotes reflect this result, one from a senior government official and the other from an academic:

“*… Generally, there is satisfaction with the performance on HR but this performance does not reach the hopeful level. Some health research conducted by academic institutions and the international agencies are valuable and with a satisfying performance. Otherwise, we need further developmental actions for better performance.*” (Gov. Expert 2)

“*… I am not satisfied with the HR performance. The production is not sufficient; students usually produce studies for degrees-related intentions, even without publishing them. A limited number of people produce research, hence, HR is not a core component in the HCS, which is not research-oriented. We have a HR unit at MOH containing 4 staff and in charge of a civil engineer officer. Even though research quality is a low and a big problem, and the gap between researcher and decision-makers is still existing without a dissemination process of knowledge which would conduct evidence for decision-makers. Moreover, the technical language of the HR outputs such as significance, T-test, Chi-Square.. etc. to be presented as policy briefs to the policy-makers who do not really know these terms in HR is a problematic issue*.” (Acad. Expert 9)

The level of satisfaction throughout IDI responses showed a wide spectrum of experts as not satisfied with HRS performance, a limited number were relatively satisfied and only a few experts were satisfied, while there were no remarkable sector variations. Pertinently, the participants responded differently about the overall performance of HRS in Palestine, where the majority of experts emphasised that it is obviously weak and still does not reach the hoped-for level. The majority of the study participants do not think that HRS is sufficiently effective and efficient, and only a limited number of experts expressed that it is effective and efficient.

“*… It is not efficient and effective because it is not well-used in the decision-making*.” (Gov. Expert 12)

“*… So, the outcomes of HR are poor, ineffective and not scientific and not from the developmental perspective.*” (Acad. Expert 9)

“*… Actually, to be fair there are many types of research that are effective but generally we face the problem of lacking a quality control and the translation process which is not applied efficiently. So I can say that the HR effectiveness and efficiency in our country are very weak and I don’t want to sound very negative but this is the fact.*” (NGO Expert 5)

It is reported that most of the perceptions across the three sectors were consistent. This can be clearly observed in the key comprehensive responses from NGOs and government perspectives. NGO experts were in line with other sectors’ views, where most of NGO experts were, to some extent, satisfied with HRS performance. Some experts indicated that it is performing quite well, where there was a variance in responses regarding the efficiency and effectiveness, with many arguing that “*we do not meet both these criteria yet*”. Some of them pointed out that there are some research or individual efforts that have met these criteria, but absolutely not as a system. An expert from United Nations Relief and Works Agency for Palestine Refugees outlined this aspect:

“*… It is improving and getting better, but it is not as active as it should be. I think it still has a long way to go. The HRS in Palestine is not yet efficient and effective, because we have so many research questions to answer.*” (NGO Expert 1)

A former senior government expert, who is involved in HR, delineated in a comprehensive sense that:

“*… Relatively satisfied with HRS performance, there is a weak conduction of clinical research, and most of them are being done for personal interests and academic degrees, they do not come from real national needs. There is no attention to research outputs. Most of the research conducted or being conducted is not derived from the actual needs of MOH, and without returning to the stated-agreed HRS priorities. The time and funding restrictions put tensions on the postgraduate students to do studies in a short time with fewer costs. Unfortunately, this makes the HRS effectiveness and efficiency almost nonexistent. Research success depends on how important that research is, and the serious problems addressed and the findings raised from the studies are not disseminated.*” (Gov. Expert 6)

### Perceptions on the political support to health research

Political attention to HRS was also received negatively with a lot of controversy and disagreement among all sectors. The following quotes reflect the overall picture of the three sectors’ perspectives, where the level of official interest in HR in Palestine is clearly weak. The first two quotes are expressed by two government officers, while the other sectors’ perspectives were almost consistent.

“*… There is an attention and it is modest from the formal level of the government, but this attention was in the past years.*” (Former Gov. Expert 2)

“*… There is no attention to the HR because we have a lack of financial support, lack of experts and resources. Donors impose their agenda on the conducting of HR.*” (Gov. Expert 9)

“*… Of course, there is attention about HRS but not as fully considered as it should be. The attention to HR from the official side is very poor*.” (Acad. Expert 5)

“*… The attention is not appropriate enough. I may say that this kind of attention is a propaganda that will not ever meet the needs of the HCS so that it can be changed and developed.*” (Acad. Expert 12)

“*… HRS is not a priority for the government. Security, politics, and infrastructure are the main priorities for our government. However, none of the projects supported researches even though they are the key to every problem we are facing. Scientific research is not our strategy.*” (NGO Expert 13)

Remarkably, responses gathered from interviews and FGDs across sectors were in harmony. Distinctively, FGDs across all sectors revealed that most FGD participants were also not fully satisfied, prominently stating in government FGDs that the research awareness and culture were not appreciated among the public health decision-makers and professionals; of course, that weakens its performance, effectiveness and efficiency according to their perceptions. Additionally, they pointed out the lack of incentive policies for researchers and decision-makers, which reflects the weakness of attention at the political level. Above all, the perceptions of the academic sector FGDs have not been optimistic, referring decisively to the absence of an effective organised body which endorses the results of executed research. This was in addition to the deficit of resources, which was seen as the most important problem. While NGO experts perceived weakness in both HR in general and the political commitment in particular, they attributed this weakness to the crumbling Palestinian entity and political power division, which led to the unconsolidated agenda and loss of agreement on HR priorities and needs alike.

### Perceptions on the gaps behind HRS performance and political attention to HR

Despite their dissatisfaction with HRS performance, government respondents strongly indicated that HR performed unsystematically; they also agreed that resources and budget deficits, weak coordination, poor knowledge dissemination and evidence utilisation and dispersant data drove their perceptions. Moreover, they described HRS as non-institutionalised into the HCS routine; the existence of donors influenced research agendas and, importantly, political attention to HRS is not sufficient. A senior government expert added that he is generally satisfied with the translation of research outputs into practice through cooperation between academic institutions and national institutes affiliated to the MOH where particular health problems are concerned.

The issues that formed the academics’ perceptions on performance, where academics were not fully satisfied, were the lack of a strategic political concern that research is conducted for academic purposes and not for social needs. The following quote comprehensively reflects most of the experts’ views:

“*… I am not fully satisfied because HR is poor, and considered as an academic requirement and based on the will of donors, where most of it is descriptive more than applied. Most of the postgraduate studies are mainly quantitative more than qualitative. Moreover, the HR is debated relating to monitoring by relevant stockholders, for example, there is a problem in the usage of health schools studies and lack of concerns by MOH to invest in those studies. Attention to HR is not adequate while it is a tool for decision-making and it is not ready enough as a system. Therefore, the outcomes will be poor, ineffective and not scientific and not from the developmental perspective.*” (Acad. Expert 1)

Moreover, three experts remarked on poor research quality as research is mostly descriptive, a shortage of resources, some stated that the unstable political conditions and the procedures of the occupation are adversely affecting it, but other experts clarified that HCS is not research oriented. The majority pointed out that the performance of research is seasonal and donor driven, while indicating that a culture aimed at improving the system performance and its efficiency and effectiveness is not promoted.

“*… HR is limited to the academics and NGOs and they do research to meet their own purposes, for example, NGOs conduct research as a way to evaluate their programs. The lack of resources influences the performance of HR. There is attention on HRS but it is not as fully considered, as it should be. The attention of the official side is very poor. Most of the HR outputs are descriptive without in-depth investigation and behind this lies the lack of funding, resources, labs, and cooperation. Studies are mostly done by individual students for academic requirements.*” (Acad. Expert 5)

The issue of the link between policy-makers and research users and coordination was raised by most participants:

“*… There is a complete disconnection between the research processes especially the academic institutes and the public sector. One of the reasons is that the research in the public sector comes from outside sources such as WHO, European Union…, so they control the field in the public sector studies. So, it is not at all effective and efficient*.” (Acad. Expert 3)

Another senior academic expert emphasised that there is no system for HR in Palestine. The expert outlined that HR performance varies greatly due to many reasons; one of them being lack of resources and that some good Palestinian researchers would be able to conduct prominent research if they were given the necessary resources. Additional thoughts delineated by this expert were mainly from a political perspective, linked to the major health problems addressed by research:

“*… There is no system. Palestinians cannot apply every single research they conduct. For example, one of the major problems that are related to health is water and environment. What can we do to solve this problem if 60% of the lands that contain water are under the Israeli occupation control? We can solve problems in health services but we cannot solve major problems. If you want me to take action, we should reject the international aid for research if it does not serve the national needs of the society. However, there is a shortage in research performance. The MOH actually knows the problems and how we can solve them but they cannot allow enough budgets to do so as many things are more important than HR.*” (Acad. Expert 16)

However, a variety of factors hinder the improvement of performance, the most prominent limitation being the unsatisfactory political interest and supportive leadership that has not yet adopted a clear vision and regulating body for HRS.

There was an identical commonality from most of the experts on the neglected role of government and other major health organisations towards HRS, which cannot be performed effectively under these circumstances. Other local NGO experts found that HRS is not a government priority, while other sectors, such as security, politics and infrastructure, have priority. Two local NGO experts illustrate these views:

“*… My satisfaction is limited where more improvement must be performed on cadre who teach scientific research. HR is not utilised in the decision and policy-making on the ground. It is supposed to be a developmental system, but I see that HRS is in a mess made by uncoordinated regulation on all levels. The system in Palestine is not completely successful; many success factors are missing. I would like to say that HRS is promising and needs support. Regarding research outputs, it is great and applicable but it was not employed in the decision-making process.*” (NGO Director 18)

The scarcity of resources, coordination and the connection between policies and researchers were a point of convergence of most experts’ views. Respondents also agreed that HR activities were or are being performed in a fragmented way and depend on wavering interest, not systematically within a clear regulating system. This means that HR activities are not commonly performed and used from development targets. Along with the quality of research, this was a crucial concern of some of the experts as expressed by this NGO expert:

“… *I perceive the HR in Palestine as weak and it needs more development and concentration on the research quality. Some researches in Palestine are strong and effective but they are few. The problem is that we miss the attention from the political leadership and this has many reasons, such as lack of financial support. For example, if research found out particular outcome or evidence, this cannot be applied due to financial resources, and there is a big problem in applied research. I think research is not always echoed into policies.*” [NGO Expert 15]

This crucial statement echoed by an international NGO expert communicates an overall understanding of the HRS, specifically reflecting HRS performance, effectiveness, efficiency and political commitment. This same participant followed with:

“… *The performance is quite good, which is based on individuals. However, structurally, HR is not that good due to governance structure in Palestine. There is an attempt to establish a council for HR such as the Palestinian National Institute of Public Health. This institute will ensure the issues of ethics, methodology, and findings and facilitating resources to the staff and researchers. I emphasise that the individual performance is amazing but systemically it is not that good. Instead, the political system, which controls the HCS, is not a good example of drawing attention to the importance of HR. We need also to find a way to effectively finance research in health. Actually, a great investment and economisation can be benefited from this system because we spend too much money on services without looking at the findings of the HR that maybe would make fewer expenses. We need also to address the way of coordination between all health providers like United Nations Relief and Works Agency for Palestine Refugees in the Near East, MOH and NGOs. This will save lots of money, guarantee user satisfaction, and improve health services. The researchers are good and they aim to improve the health service but these researches are not organised.*” (International NGO Expert 12)

## Discussion

In this study, we aimed first to explore the satisfaction of experts across three sectors involved in HR in Palestine on the overall HRS performance. Second, we investigated their perceptions about the state political interest and commitment to Palestinian HRS. Third, we identified the actual gaps behind system underperformance and lack of official governmental support. Generally, the overall HRS performance in Palestine is perceived to be considerably low. Therefore, the satisfaction pattern was relatively paradoxical; whereas the academics and NGO experts were comparatively satisfied, very few of their government counterparts were fully satisfied. Additionally, the majority of experts found HRS to be ineffective and inefficient.

We reached these findings through analysis of interviews and FGDs with stakeholders that often influence and lead this system. A well-functioning national research system requires a holistic understanding of the system’s conceptual components and performance [4, 14]. Ensuring a well-performing HRS supported by an official state commitment is essential [15], because governments and donors are increasingly interested in evaluating the benefits of their investments in HR [5].

The strengthening of HRS is key to meeting national health and economic needs, particularly the performance component, to monitor and evaluate system operations. Performance frameworks may consist of indicators and models, agreed upon nationally and built in the HCS structure, for systematic measurements [4]. Besides using those conceptual frameworks, developed by international bodies, to assess HRS performance by compiling certain measurements [5, 14, 22, 27, 34], a combined analysis is an additional approach that could be useful in understanding the performance of HR from different aspects. Furthermore, using practical tools to measure HRS performance should be technically recognised by system stakeholders on the one hand, yet understanding their views is crucial on the other. For this, integrating both approaches might better support an accurate understanding from a system perspective; nevertheless, such an understanding perspective is lacking [5]. This work represents only a modest development attempt by employing a descriptive perception analysis to realise the system processes and gaps to be strengthened.

Our study finds that research performance measurements in Palestine, whether quantitative or qualitative, are not established. COHRED found that few Middle Eastern countries have a system of monitoring and evaluation for its HRSs [34]. Therefore, the study assumes that there is no HRS, as this concept is an emergent one and not fully conceptualised or appreciated [21, 35, 36]. The lack of monitoring and evaluation for HRSs raises two concerns; first, it means that HR is non-institutionalised into HCS and, second, it indicates a lack of stewardship. A study supported our findings that continuous monitoring and evaluation is required to ensure efficient resource use based on agreed priorities and appropriately conducting research in an ethical manner. It also clarified that assessing HRS governance, which performs these tasks, will provide a broader picture of national HRS capacity and performance [37].

The results of this study are supported by findings from several other studies [17, 23, 38] identifying relevant factors that result in HRS underperformance. These factors can be considered as problematic gaps that lead to low HR performance in Palestine. A lack of awareness and an unappreciated culture on HR, as proven by a national study [26], as well as the lack of incentive policies for researchers and decision-makers, were two of these factors. Moreover, an effective organising body to take over the duty of research evidence embeddedness into decision- and policy-making is absent, and therefore research translation is not significantly applied in Palestine and most Middle Eastern countries [36]. In fact, there is no country in the Middle East reporting systematic efforts to feed research results into decision-making in the health sector [35]. Yet, cultivating and improving an evidence-based culture and practice is crucial [39]. Other major factors for system underperformance were a shortfall of resources and missing political will, which was seen as an obstacle throughout the Middle East region [2]. Both attributed to the weakness of the crumbling Palestinian entity due to the Israeli occupation and internal political division. This causes an unconsolidated agenda, disagreed HR priorities and needs, and eventually the wasting of resources in this donor-dependent country.

Additional stated factors include that research activities are seasonal, namely that they are geared by the donor and solicited by the Palestinian researchers’ personal interests. Moreover, these activities are unguided developmentally and individual driven, while COHRED considered HRS as an approach for achieving sustainable development [24, 28, 40]. It is reported that research addresses academic purposes rather than society needs, which are not used for health decisions. Other literature stressed that HR is one of the main driving forces for improving the performance of health systems and ultimately the health of populations, as well as crucially needed to attain and track the achievements of the United Nations Millennium Development Goals [5].

Fractured, non-participatory coordination among stakeholders in knowledge production and data dissemination is also assumed to be a leading problem that results in underperformance and system frustration in achieving the desired goals [10, 21, 41]. Lack of system performance means that evidence is not often utilised in decision-making, even in the Eastern Mediterranean Region countries [42, 43]. In addition, the quality of research produced by many Palestinian institutions is not satisfactory [44].

HR is obviously absent from the agenda and does not get attention at the official political level, yet political will and commitment is a necessary factor, as described by WHO in its strategy on research for health [3]. Most of the experts highlighted a lack of strategic political concern, where research is not a priority and legitimately embraced. Additionally, there was an identical commonality from most of the experts on the neglected role of government and other major health organisations towards HRS. The Palestinian government, and the MOH in particular, did not distinctly indicate health or scientific research as inherent components in neither its national agenda 2017–2022 nor in its central budgets [45, 46]. This means that the Palestinian official concern basically focuses on security, politics, crises management and service-sustained systems due to the exceptional political and security situation. Therefore, the government concern is intermittent and does not come in the context of a constant national perspective, which may also be reflected at the institutional level.

However, a variety of factors hinder the improvement of performance, the most prominent limitation expressed being unsatisfactory political interest and unsupportive leadership that has not yet adopted a clear vision and regulating body for HRS.

Our study strengths include (1) it being the first HRS descriptive research conducted in Palestine; (2) the sampling of a very diverse group of stakeholders across three sectors, including policy-makers, academia and representatives from local and international NGOs; (3) the use of IDIs and FGDs based on internationally developed frameworks; (4) the focus being primarily on the policy level of the HRS and system understanding; and (5) the fact that the study could be a significant basis for the national and international bodies in any upcoming strengthening endeavours such as MOH, WHO and COHRED. The study limitations included (1) a lack of sufficient and up-to-date reports and data on the HR components, as well as a lack of literature, particularly investigating the perceptions of system players; (2) research team movement was restricted in the field; and (3) the unavailability of high seniors due to time limitations, and therefore the lack of involvement of more leadership levels across sectors. Further, some IDIs and FGDs were shorter than expected and some questions were insufficiently answered due to a lack of knowledge, practices and time constraints.

## Conclusion

HR in Palestine is progressing, despite the unprecedented conditions, instability and fragility. However, there remain substantial windows of opportunity for actions to make positive changes in the HR structure and performance. Nevertheless, without systematic assessment and mapping mechanisms, HRS performance will remain below the satisfactory level. Several factors behind system underperformance have been recognised; first, the weakness of the research culture within institutions, and the lack of political will and serious adoption and support. Secondly, research activity is individualistic, non-development oriented and non-invested in decision-making, with a fragility of coordination. Finally, the severe shortage of resources and, therefore, capacity.

Due to the serious insufficiency of literature in the local and regional levels regarding assessment of HRS performance, it is very important to intensify further efforts to assess the performance of HR in Palestine using internationally adopted analysis frameworks. On the other hand, it is also valuable to conduct national studies to realise the impact of HR on the HCS and society alike.

In general, HR is neither ineffective nor efficient; however, serious development actions should be taken in order to establish integrated and well-functioning system components. In this respect, study findings can help inform and steer future plans and activities for the Palestinian health decision-makers in contributing to the development of not only the research performance assessment, but also the other system components to be cohesively structured and successfully functioning. This study proposes various policy development insights related to system performance in particular and other system pillars combined.

These suggestions depend on a myriad of actions that need to be shared on a basic level with Palestinian HCS policy-makers and seniors. First and foremost is the availability of political decision and willingness from the three sectors’ leaderships with the support of international partners. Official political concern can be encouraged through political interaction, policy dialogs and advocacy campaigns. In doing so, shaping governance structure and building a national body for HR unifying all relevant stakeholders is essential. This body should formulate a national policy dedicated to HR; one of these policy components requires focusing on HR performance issues to be inherently promoted. This policy should focus on (1) actions to address the deficiency of research awareness and culture, as a philosophy and practice, among all health professionals through regular awareness and orientation actions; (2) a serious emphasis on tackling the lack of skills and capabilities by implementing systematic capacity-building and educational programmes targetting the decision- and policy-makers on the topic of HR assessments; and (3) reducing unsystematic and individualistic research efforts, HR needs to be institutionalised and functionally performed from a development perspective, as well unified in an interdisciplinary and well-coordinated manner. This should be based on established and agreed-upon performance guidelines, whether qualitative or quantitative, to be integrated institutionally and nationally. The guidelines or monitoring and evaluation measurements can be taken from developed international frameworks for HRS, concurrently seeking to establish a national observatory platform for HR, led by the MOH and academia, in order to assess the three phases of HR (financing, production and utilisation) and to track research trends in terms of quality, quantity, relevance and impacts.

## Additional file

Additional file 1: **Supplement 1.** Map of Palestine. **Supplement 2.** List of targeted institutions across the government, academic universities, and local and international non-governmental organisations working in Palestine. **Supplement 3.** Selection criteria for selected study institutions and participants. **Supplement 4.** The study instruments (in-depth interviews (IDIs) and focus group discussions (FGDs)). **Table S1.** Characteristics of the IDI participants. **Table S2.** Characteristics of the FGDs participants. (DOCX 79 kb)

## Abbreviations

COHRED: Council on Health Research for Development
FGDs: focus group discussions
GS: Gaza Strip
HCS: healthcare system
HR: health research
HRS: health research system
IDIs: in-depth interviews
MOH: Ministry of Health
NGOs: non-governmental organisations
WB: West Bank
