# Supplementary material for: Assessing policy-makers’, academics’ and experts’ satisfaction with the performance of the Palestinian health research system: a qualitative study
Source: Health Res Policy Syst. 2018 Jul 25;16:66. doi: 10.1186/s12961-018-0341-x (PMC6060523; doi:10.1186/s12961-018-0341-x)
Supplement: Supplementary file 1 — Supplement 1. Map of Palestine. Supplement 2. List of targeted institutions across the government, academic universities, and local and international non-governmental organisations working in Palestine. Supplement 3. Selection criteria for selected study institutions and participants. Supplement 4. The study instruments (in-depth interviews (IDIs) and focus group discussions (FGDs)). Table S1. Characteristics of the IDI participants. Table S2. Characteristics of the FGDs participants. (DOCX 79 kb) [file 12961_2018_341_MOESM1_ESM.docx]

**Supplements of the 2nd Paper**

**Assessing the satisfaction pattern of policymakers, academics, and experts towards the performance of Palestinian HRS: A qualitative study**

**Content:**

1. **Supplements**

**Supplement 1: Palestine map**

**Supplement 2: List of targeted institutions across the government, academic universities and local and international NGOs who working in Palestine**

**Supplement 3: Selection criteria for selected study institutions and participants**

**Supplement 4. The study instruments (IDIs and FGDs)**

1. **Tables**

**Table S1: Characteristics of the IDI Participants**

**Table S2: Characteristics of the FGDs Participants**

**Supplement (1): Palestine map**


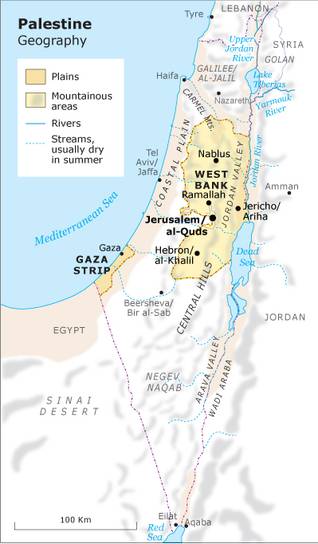


| **Targeted Government Authorities in Palestine** | | | | |
| --- | --- | --- | --- | --- |
| **No.** | **Org. abbreviation** | **Full name** | **No. of IDIs Participants** | **No. of FGDs Participants** |
| 1. | MOH | Former MOH Minister and Deputy Minister | **2** |  |
|  |  | General Directorate (GD) of Hospitals |  | 2 |
|  |  | GD of Primary Care | 1 | 1 |
|  |  | GD of Health Information | 1 | 2 |
|  |  | GD of International Cooperation |  | 1 |
|  |  | GD of Pharmaceuticals and Medical Supplies |  | 1 |
|  |  | Department of Surgeries at General Directorate of Hospitals |  | 1 |
|  |  | Department of Health Research | 1 |  |
|  |  | Department of Cath. and Cardiac Care at General Directorate of Hospitals |  | 2 |
|  |  | Department of Public Health |  | 1 |
|  |  | Department of Education |  | 1 |
|  |  | Department of Health Economic |  | 1 |
|  |  | Department of Strategic Planning | 2 | 1 |
| 2. | MOFP | Ministry of Finance and Planning |  | 2 |
| 3. | MOHE | Ministry of Higher Education | 2 | 2 |
| 4. | PLC | Palestinian Legislative Council | 3 |  |
| 5. | PCBS | Palestinian Central Bureau of Statistics | 2 | 1 |
| 6. | PMC | Palestine Medical Council | 1 |  |
|  |  |  | **15** | **19** |

**Supplement (2), List of Targeted Institutions across the Government, Academic Universities and Local and International NGOs who working in Palestine**

| **Targeted Academic Institutions in Palestine** | | | |
| --- | --- | --- | --- |
| **No.** | **Institution Name** | **No. of IDIs Participants** | **No. of FGDs Participants** |
| 1. | Birziet University, Institute of Community and Public Health | 3 | 3 |
| 2. | Al-Quds University, School of Public Health | 5 | 1 |
| 3. | Annajah National University, Faculty of Medicine and Health Sciences | 3 | 2 |
| 4. | The Islamic University of Gaza, Faculty of Medicine, Faculty of Nursing | 2 | 2 |
| 5. | Al-Azhar University, Faculty of Applied Medical Sciences | 2 | 1 |
| 6. | Al-Aqsa University, Faculty of Applied Sciences |  | 1 |
| 7. | The University of Palestine, Faculty of Health Professions |  | 2 |
| 8. | Arab American University, Faculty of Health Sciences | 1 |  |
| 9. | Palestine College of Nursing | 1 |  |
| 10. | University College of Applied Sciences, Department of Applied Health Sciences |  | 1 |
| 11. | The American University of Beirut, Faculty of Health Sciences | **1** |  |
|  |  | **18** | **13** |

| **Targeted international organizations in Palestine** | | | | |
| --- | --- | --- | --- | --- |
| ***No.*** | ***Organization abbreviation*** | ***Full name*** | ***No. of IDIs Participants*** | ***No. of FGDs Participants*** |
| 1. | UNRWA | The United Nations Relief and Works Agency for Palestine Refugees in the Near East (UNRWA) | 2 | 2 |
| 2. | WHO | World Health Organization, Palestinian National Institute of Public Health | 2 |  |
| 3. | UNICEF | The United Nations Children's Fund | 1 | 1 |
| 4. | UNFPA | United Nations Population Fund | 2 |  |
| 5. | W.V. | World Vision |  | 2 |
| 6. | SC | Save the Children |  | 1 |
| 7. | MDM | Médecins du Monde |  | 1 |
| 8. | MAP-UK | Medical Aid for Palestinians |  | 1 |
| 9. | MC | Mercy Corps | 1 |  |
| 10. | QC | Qatar Charity |  | 1 |
|  |  |  | **8** | **9** |

| **Targeted Local organizations in Palestine** | | | | |
| --- | --- | --- | --- | --- |
| **No.** | **Organization abbreviation** | **Full name** | **No. of IDIs Participants** | **No. of FGDs Participants** |
| 1. | PFPPA | The Palestinian Family Planning and Protection Association | 1 | 2 |
| 2. | PRCS | Palestinian Red Crescent Society | 1 | 1 |
| 3. | HDIP | Health, Development, Information and Policy Institute |  | 1 |
| 4. | UHWC | Union of Health Work Committee | 1 |  |
| 5. | UHCC | Union of Health Care Committees | 2 | 2 |
| 6. | PMRS | Palestinian Medical Relief Society | 1 | 2 |
| 7. | AEPBA | Ard El Insan Palestinian Benevolent Association | 1 | 1 |
| 8. | Pal-think | Pal-think Centre for Strategic Studies | 1 |  |
| 9. | GCMHP | Gaza Community Mental Health Programme | 1 |  |
| 10. | WA | Welfare Association | 1 |  |
| 11. | JUZOOR | JUZOOR for Health & Social Development | 1 |  |
|  |  |  | **11** | **9** |

**Supplement (3). Selection criteria of selected study institutions and participants**

**Inclusion criteria**

- ***Institutional level (sectors)***

1. **Academic**

- To be official institutionalized status actually operates and regularly produces health research.
- It must be academic institutions officially registered; national or private will be included.
- Has a role and contribution in the health sciences and public health research production.
- Has health school, which offers graduate or postgraduate health specialties (public health, health management, epidemiology, environmental health, maternal child health, community mental health, nutrition, biology, laboratory, or any other relevant specializations).

1. **NGO**

- To have institutionalized and licensed entity, and actually operates.
- A non-profit organization and independent from the government.
- Not inherited, membership is voluntary, and is not based on blood or tribe.
- Works basically in the health scope and fully involved in producing and using health research in one of the targeted health research fields.
- The study considered the NGO sector as an including the health local Palestinian NGOs, international NGOs and private institutes which are essentially engaged in the health research actions in Palestine whether producer, users or funders.

1. **Government**

All of the relevant governmental ministries, including MOH, MOHE, MOFP, PLC, PMC and PCBS, those bodies are mainly involved in health sciences and public health, and they are in charge of using research findings into policies, as well as funding the research. The following departments at MOH also will be given the most attention in this study as illustrated in complement (1). Moreover, the health committee at the PLC and any other national committee such PMC will be also embraced.

- **Individual level**

***The study participants from the targeted three sectors were based on these criteria:***

From the academic sector, all of faculty members and researchers within the targeted faculties who are officially working and enrolled in the interesting research activities will be selected. At the government sector, all of legislators and experts from the national council and committee, policy and decision makers and directors of departments will be involved. The participants from the NGO, private and international agencies sector, organization’s directors, researchers, and local, regional and international experts alike will be selected.

**Exclusion criteria**

The study participants at the institutional and individual levels who do not meet the above criteria will be excluded.

**Supplement (4). The study Instruments (IDIs and FGDs)**

**Supplement (4a) In-depth Interview Questions**

**Introduction**

Thanks for participating in this interview ….warm greetings, interviewer introduce himself, and explain briefly the purpose and nature of the interview, as well as its agendas and topics.

**Socio-demographic ccharacteristics:**

Age:_____ Gender:_______ Level of education: __________________Year of experience: ______ Sector affiliation: __________________ Location: ________________ Position: ______________

Contact: __________________________

1. **HRS conceptualization and HRS performance**

As we are interested in the way our interviewees define HRS (rather than starting the interview with our own definition of it), we will ask all interviewees about how they perceive health systems research.

- **Conceptualization**
- From your own definition, how do you define the HRS?
- How do you perceive the HRS in Palestine?
- When we mention health research in Palestine, what comes to your mind first?
- What we gain from HRS and what we will lose by giving that up?
- **HRS performance**
- From your perspective, are you pleased with how health research is performed, produced and used? Explain?
- From your perspective, is the attention of HRS is appropriate (developmental versus occasional)?
- Do you think that the HRS in Palestine is effective and efficient? Why?

1. **HRS governance, policy, and priorities**

- **Governance**
- Describe the governance structure or health research in Palestine?, who govern the system (bodies), manage the relationships, processes, and rules for making a decision within the system.
- Describe the ethical review processes or structures for health research in Palestine?
- **Policy**
- Based on your knowledge and experience, is there a national policy that managing the work of health research? Please justify?
- Where the problems that are related to health research policy lies at the three sectors government, academia, and NGOs?
- What could be done to boost the improvement of HRS policies?
- **Priorities**
- From your views, are there national priorities for health research? and does it constantly identified in accordance with the needs?
- Do not you think that health research which was applied and that still applies deriving from national health priorities and based on people needs?, and How do you prove that?
- What are the most important HRS needs and priorities for improvement?

1. **Health research stakeholders**

- Who are essential relevant parties should be engaged in health research field?
- What are the roles for each of them respecting to research producer and user?
- How do you evaluate these roles? And are they carrying out their responsibilities properly?
- In Palestine, what is the role of international agencies in supporting HRS? and what are the ways to accomplish that?
- How do you find the relationship and cooperation among the HRS stakeholders? What are the most appropriate interventions to develop it?

1. **HRS capacities, resources, knowledge transfer and application, and quality**

- Describe the actual HRS capacities at the institutional and individual levels?
- Do you think that there is a need to develop those capacities? how, and what are the priorities to attain this?
- Tell us the mechanism of funding the health research in Palestine, whether it is sufficient and sustainable? what is the hoped?
- Explain the reality of human resources in health research? and what further development opportunities in this direction?

**Knowledge transfer and translation**

- Are you satisfied with the way of knowledge dissemination among research producer and user?
- What is required to improve and promote communication and networking between research producer and user?
- How do the research translation process work and what role does research play in decision-making?
- What could be done to boost the improvement of HRS policies and use of evidence in health policy decision making?

**HRS versus standardization**

- Does the health research in Palestine meet the international standardization? , why, and what is required to achieve the highest level of its quality?

1. **HRS pitfalls and challenges, and insights for HRS strengthening**

- **HRS strengths, weaknesses, opportunities and threats (SWOT)**
- Could you summarize the HRS in Palestine in reference to SWOT?
- **HRS related to supporting health system performance and health outcomes**
- How is the health research role in improving the performance of the health system and national health indicators?
- **HRS challenges and obstacles**
- From your perspective, what are the challenges facing the health research in Palestine?
- Individual-level - Institutional-level - National-level
- **How can the debate around HRS in Palestine among stakeholders be reduced? What should be done?**
- **Based on your understanding, what you suggest recommendations and developmental actions for better production and utilization of HRS in the PHCS components at the institutional and individual levels, in order to support sustainable health development?**

**Additional comments, is there anything else you would like to share with us or any questions you have for us?** (This question already starts the debriefing, and the interviewer can even engage in a discussion here).

**The end …**

**Supplement (4b). Focus group discussion FGD**

***Focus group structure and protocol***

- **Preparation and reception**
- **Session information**

Participant affiliation: ______________________

Number of participants: ____________________

Place/location: ___________________________

Time: start ____________ end _______________

Date: ________________

- **Introduction**

Hello, my name is Mohammed Al-Khaldi. Thank you for taking the time to participate in a focus group on the needs of investigating the health research situation in Palestine. This focus group is part of a larger research project process that Swiss TPH is conducting to learn about the Health Research System in Palestine. We want to understand its status properly, and to identify the strengths, weaknesses, opportunities and threats. Moreover, how we might promote it for better production and utilization.

We would like to hear from you about the health research status generally from the side, and institutionally from the other side, and also your perspectives you would suggest in reference to the particular topic components. During this focus group, I will ask questions and facilitate a conversation and discussion about this topic. Please keep in mind that there are no “right” or “wrong” answers to any of the questions I will ask. The purpose is to stimulate conversation and hear the opinions of everyone in the room. I hope you will be comfortable speaking honestly and sharing your ideas with us.

Please note that this session will be recorded by assistant will be taking notes during the focus group to ensure we adequately capture your ideas during the discussion. However, the comments from the focus group will remain confidential and your name will not be attached to any comments you make. We would like to emphasize on the interaction and active participation between or among group members. Do you have any questions before we begin?

- **Focus group themes for discussion**
- **Theme 1:** Overall understanding about HRS concept, goals, functions and performance in Palestine
- **Theme 2:** HRS governance, policy, and priorities
- **Theme 3:** Stakeholders and coordination status
- **Theme 4:** HRS capacities, knowledge transfer, dissemination and quality, and evidence application
- **Theme 5:** Identification of HRS SWOT, challenges, and perspectives for improvements

**Theme 1: Overall understanding and performance about HRS reality**

- **Conceptualization**
- When we mention the health research, how do you perceive this concept? what it means?, and what are the most important elements joined with it?
- Let’s do a quick round to the health research landscape, give us a comprehensive image about health research and its climate in Palestine?
- Where we are now, and are you satisfied with HRS in Palestine? why?
- **Performance**
- Is the health research performs appropriately in a right task? Are its activities achieving the goals?
- Is it manage and implement in an optimal way?
- How do you see the political commitment from policymakers and legislators toward HSR?, what is your explanation for that?

**Theme 2: Governance, policy, and priorities**

- **Governance**
- Describe the governance structure or health mechanisms in Palestine?, who govern the system (bodies), manage the relationships, processes, and rules for making a decision within the system?
- Please, can we discuss collectively the health research governance, structure, and its management processes?.
- Describe the ethical review process or structure for health research in Palestine?
- **Policy**
- Are there clear and effective policies for health research at the level of your institution and at the national level as a whole?

**If any,** what is your evaluation of such policies, and what needed to be strengthened?

**If does not exist,** how do you interpret that?

- In the national health strategy or institutional strategic plan, are there items focuses on strengthening health research?, can you justify that?
- **Priority/needs**
- What are the national health priorities in Palestine?, at your institution level, what are the health priorities? Are both priorities formulated regularly?
- Is there a match between those priorities and the national health research priorities?
- What are the national health research priorities?, and how are the health research priorities and agendas determined at present?

**Theme 3: Stakeholders and coordination status**

- Who are the major actors in health research in Palestine? what are the roles of each of them? How do you evaluate their roles?
- How do you describe the status of cooperation and coordination pattern for HRS in Palestine?.
- What mechanisms exist for coordinating health research? And what are there mechanisms for avoiding duplication?

**Theme 4: HRS capacities, knowledge transfer, dissemination and quality, and evidence application**

- **Capacities and resources**
- How do you mobilize resources for research at present/ what are the existing resource mobilization strategies?

1. What have been the challenges?
2. If any challenges, how have you addressed these?
3. What can/could have been done differently?
4. Who finances the HRS?, and What is the government role in allocating resources for health research?

- Have there been any challenges, in terms of budgeting, allocation, and utilization of funds in the area of (health) research?
- How might the government increase its budget allocation to (health) research?
- Are there any other national and international sources of funding for (health) research in Palestine? What is the role of external financing in this context?
- In terms of institutional and human resources and capacities, what is the existing capacity for (health) research? Are there any mechanisms or opportunities for capacity strengthening or training initiatives in health research?

**If, yes, what are these? , If no, why not?**

- Do the universities have the capacity for producing researchers? What about in terms of health research? (How)

1. What is being done currently?
2. Are there any international collaborative relationships for training researchers? Who are the major actors?
3. What is your perception of these relationships?
4. What could be done differently?

- **Technically, In your opinion, what do you think the health research practice across the producer and user in reference to:**
- **Quality and standardization**
- Explain how do you perceive the quality of health research production?
- Does it meet the international guidelines?
- **Knowledge dissemination and evidence translation**
- Are there mechanisms for ensuring effective management and use of knowledge in Palestine?
- What opportunities exist for management and use of knowledge?
- Are there any challenges in the management and use of knowledge?
- In what ways and how could use of generated knowledge be improved?
- Are researchers required to deposit papers, findings etc. into a database/central repository?
- Do health researchers have access to the knowledge produced in Palestine?
- Do health providers, policymakers, and managers have access to the knowledge produced in Palestine?
- How could you evaluate the KT?, Are there any mechanisms/ systems in place aimed at ensuring increased access to knowledge?
- How could you evaluate the Knowledge application?, How or what mechanisms exist to ensure that research addresses the questions of policy makers?)
- Is there a database for storage of research findings, projects, and reports?
- Who is responsible for disseminating and translating health research knowledge?
- What can/could have been done differently to promote these practices?

**Theme 5: Identification of HRS SWOT, challenges, and perspectives for improvements**

- **SWOT**
- What are the prominent strengths and weaknesses of the health research in Palestine?
- Looking for the future, could you determine the opportunities to be promoted and threats to be tackled regarding health research development in Palestine?
- **Recognition the HRS challenges and gaps in Palestine.**
- Let us focus on challenges, can you summarize the health research gaps and constraints in Palestine at the: a) National level, b) Institutional level and c) Individual level
- **Recommendations for changes and improvements**
- Based on your understanding, what you suggest to strengthen the Palestinian HRS production and utilization at the: government, academia, local and international NGO sectors
- **Is there anything else we haven’t discussed yet that you think is important about health research in Palestine, or additional comments would like to add on this theme and the others?**

**The end**

**Table S1: Characteristics of the IDI Participants**

| **Ch.ch.**  **Sector** | **Age** | | | **Gender** | | **Education level** | | | **Experience years** | | |  | **participants per locations** | | | | |
| --- | --- | --- | --- | --- | --- | --- | --- | --- | --- | --- | --- | --- | --- | --- | --- | --- | --- |
|  | **30-50** | **51-60** | **>60** | **F** | **M** | **BA/Dip** | **MA** | **PhD** | **<10** | **10-20** | **>20** |  | **WB** | **GS** | **Leb.** | **Jor.** | **Egy.** |
| **Acad.** | **7** | **7** | **4** | **5** | **13** |  |  | **18** |  | **11** | **7** |  | **10** | **7** | **1** |  |  |
|  | **Leadership positions** | | | | | | | | | | |  | **No. of institutions vs locations** | | | | |
|  | **VP** | **Dean** | | **VD** | | **HRD** | | **Ass. Prof.** | | **Assis. Prof.** | |  | 4 | 3 | 1 |  |  |
|  | **1** | **5** | | **2** | | **2** | | **6** | | **2** | |  | **Participants: 18, Institutions: 8** | | | | |
|  | | | | | | | | | | | | | | | | | |
| **Gov.** | **30-50** | **51-60** | **>60** | **F** | **M** | **BA/Dip** | **MA** | **PhD** | **<10** | **10-20** | **>20** |  | **No. participants and institutions vs locations (alike)** | | | | |
|  | 5 | 7 | 3 | 3 | 12 | 4 | 6 | 5 | 1 | 6 | 8 |  | **WB** | **GS** | **Leb.** | **Jor.** | **Egy.** |
|  | **Leadership positions** | | | | | | | | | | |  | 11 | 4 |  |  |  |
|  | **FM/DM** | **NHM** | | **GD** | | **Director** | | **HD** | |  |  |  | **Participants: 15**  **Institutions: 5 and 9 departments** | | | | |
|  | **3** | **4** | | **1** | | **3** | | **4** | |  |  |  |  |  |  |  |  |
|  | | | | | | | | | | | | | | | | | |
| **NGOs** | **30-50** | **51-60** | **>60** | **F** | **M** | **BA/Dip** | **MA** | **PhD** | **<10** | **10-20** | **>20** |  | **No. of participants vs locations** | | | | |
|  | **11** | **8** |  | **6** | **13** | **1** | **13** | **5** | **3** | **4** | **12** |  | **WB** | **GS** | **Leb.** | **Jor.** | **Egy.** |
|  | **Leadership positions** | | | | | | | | | | |  | 10 | 7 |  | 1 | 1 |
|  | **ED** | **Director** | | **HO** | | **CO** | | **PO** | |  |  |  | **Participants: 19**  **Institutions: 15, 10 local NGOs and 5 INGOs** | | | | |
|  | **5** | **3** | | **2** | | **4** | | **5** | |  |  |  |  |  |  |  |  |
| **Sectors:** **Acd** : academic, **Gov** : government, **NGOs**: includes local and international non-governmental organizations  **Gender:** **F**: female, **M**: male  **Education:** **BA/Dip**: bachelor and diploma, **MA**: master, **Ph.D.**: doctor of philosophy  **Location:** **WB**: west bank, **GS**: Gaza Strip, **Leb**: Lebanon, **Jor**: Jordan, **Egy**: Egypt  **Position:** **VP**: vice president, **D**: dean, **VD**: vice dean, **HRD**: head of the research department, **Asso. Prof**: Associate professor, **Assis. Prof**.: assistant prof.  **FM/DM** : former minister or deputy minister, **NCM**: national council member, **GD**: general director, **HD**: head of department,  **ED**: executive director, **HO**: head of the office, **CO**: chief officer, **PO**: program officer | | | | | | | | | | | | | | | | | |

**Table S2: Characteristics of the FGDs Participants**

| **Ch.ch**  **Sector** | **Age** | | | **Gender** | | **Education level** | | | | | | **Years of experience** | | | **Total** | | |
| --- | --- | --- | --- | --- | --- | --- | --- | --- | --- | --- | --- | --- | --- | --- | --- | --- | --- |
|  | **30-40** | **41-50** | **>50** | **F** | **M** | **BA/Dip** | | | **MA** | | **PhD** | **< 10y** | **10-20y** | **>20y** | **Participants (14)** | | **Institutions (8)** |
|  |  |  |  |  |  |  |  |  |  |  |  |  |  |  | **WB FGDs** | **GS FGDs** |  |
| **2 Acad. FGDs** |  | **7** | **7** | **3** | **11** |  | | |  | | **14** | **4** | **6** | **4** | **6** | **8** |  |
|  | **Leadership position** | | | **D** | | | **FP** | | | | | **Asso. Prof.** | | | **Assi. Prof.** | | |
|  |  |  |  | **1** | | | **1** | | | | | **5** | | | **7** | | |
|  | | | | | | | | | | | | | | | | | |
| **2 Gov.**  **FGDs** | **30-40** | **41-50** | **>50** | **F** | **M** | **BA/Dip** | | | **MA** | | **PhD** | **< 10y** | **10-20y** | **>20y** | **Participants (20)** | | **(18):**  **4 institutions**  **14 departments** |
|  |  |  |  |  |  |  |  |  |  |  |  |  |  |  | **WB FGDs** | **GS FGDs** |  |
|  | **2** | **8** | **10** | **5** | **15** | **1** | | | **10** | | **9** |  | **13** | **7** | **12** | **8** |  |
|  | **Leadership position** | | | **NCM** | | | **GD** | | | | | **D** | | | **HD** | | |
|  |  |  |  | **1** | | | **8** | | | | | **5** | | | **5** | | |
|  | | | | | | | | | | | | | | | | | |
| **2 NGO FGDs** | **30-40** | **41-50** | **>50** | **F** | **M** | **BA/Dip** | | **MA** | | | **PhD** | **< 10y** | **10-20y** | **>20y** | **Participants (18)** | | **(13):**  **6 LNGO**  **7 INGO** |
|  |  |  |  |  |  |  |  |  |  |  |  |  |  |  | **WB FGDs** | **GS FGDs** |  |
|  | **3** | **12** | **3** | **7** | **11** | **3** | | **15** | |  | **2** | | **8** | **8** | **10** | **8** |  |
|  | **Leadership position** | | | **ED** | | | | | | | **PM** | | | | **SO** | | |
|  |  |  |  | **5** | | | | | | | **8** | | | | **5** | | |
| **FGDs**: focus group discussions  **Sectors:** **Acd** : academic, **Gov** : government, **NGOs**: includes local and international non-governmental organizations  **Gender:** **F**: female, **M**: male  **Education:** **BA/Dip**: bachelor and diploma, **MA**: master, **Ph.D.**: doctor of philosophy  **Location:** **WBFGDs**: west bank FGDs, **GSFGDs**: Gaza strip FGDS  **Position:** **D**: dean, **FP**: full professor, **Asso. Prof**: Associate professor, **Assi. Prof**.: assistant prof. **NCM**: national council member, **GD**: general director, **D**: director, **HD**: head of the department, **ED**: executive director, **PM**: program manager, **SO**: senior officer | | | | | | | | | | | | | | | | | |
